# Spontaneous regression rates of actinic keratosis: a systematic review and pooled analysis of randomized controlled trials

**DOI:** 10.1038/s41598-022-09722-8

**Published:** 2022-04-07

**Authors:** Theresa Steeb, Anne Petzold, Annkathrin Hornung, Anja Wessely, Carola Berking, Markus V. Heppt

**Affiliations:** 1 Department of Dermatology, Universitätsklinikum Erlangen, Friedrich-Alexander-University Erlangen-Nürnberg (FAU), Ulmenweg 18, 91054 Erlangen, Germany; 2 Comprehensive Cancer Center Erlangen-European Metropolitan Area of Nuremberg (CCC ER-EMN) and Deutsches Zentrum Immuntherapie (DZI), Erlangen, Germany

**Keywords:** Skin diseases, Squamous cell carcinoma

## Abstract

Actinic keratosis (AK) are precancerous lesions of the skin which may progress to invasive squamous cell carcinoma. However, single lesions may also persist or even regress and heal spontaneously. Until now, evidence on the natural course of AK including spontaneous regression is limited. We aimed to synthesize regression rates of AK. We performed a systematic literature research in Medline, Embase, and CENTRAL for eligible trials until 3rd March 2020. Spontaneous regression rates were pooled using a random-effects model to calculate pooled proportions of participant-specific and lesion-specific complete clearance rates reported for the placebo arms of randomized controlled trials. Subgroup analyses were performed to dissect differences according to the type of placebo, immunocompetence of the participants, and localization of the lesions. Data from 38 records was included. The pooled participant-specific clearance rate was 8% (95% CI 6–10%, I^2^ = 71%) while the lesion-specific clearance rate was 23% (95% CI 16–31%, I^2^ = 97%). The highest participant- and lesion-specific clearance rates were achieved 12 weeks after the end of treatment (12% and 33%, respectively). Subgroup analysis revealed participant- as well as lesion-specific clearance rates of 0% for organ transplant recipients (OTR). We conclude that only a few participants achieve complete regression of their AK without any active treatment. Besides, the results underline that lesion clearance without active treatment is unlikely in OTR. Thus, early and consequent treatment of AK is recommended. Special attention should be paid when treating AK of OTR.

## Introduction

Actinic keratoses (AK) are common precancerous lesions of the skin which are induced by long-term exposure to ultraviolet (UV) radiation^1,2^. They usually present as erythematous and keratotic or scaling patches with a rough, sandpaper-like surface on chronically sun-exposed areas such as the face, ears, arms, and dorsal hands^2,3^. Due to their prevalence of up to 60% in light-skinned individuals over the age of 60 years, they represent one of the most common skin lesions^4,5^. Furthermore, they are among the most common conditions treated by dermatologists in Western countries^4,6^. As a result of an aging population as well as increased exposure to UV radiation due to a change in leisure behavior and outdoor activities, the incidence has been steadily increasing globally over the past years^5,7^.

AK may progress into invasive cutaneous squamous cell carcinoma (cSCC). However, reliable biomarkers to predict whether a lesion will transform to invasive cSCC or not are still lacking. Nevertheless, the transformation risk is estimated to be low for single lesions^8^. Progression rates of AK to cSCC have been found to range between 0 and 0.075% per lesion-year, with a risk of up to 0.53% per lesion in patients with a prior history of non-melanoma skin cancer^9^. However, if multiple AK are present and accompanied by signs of chronic actinic damage or field cancerization, the risk of malignant conversion increases rapidly. To this end, the progression rates are hard to determine due to the long latency of cSCC formation and continuously intervening treatments for AK as a chronic health condition^9,10^. Thus, international guidelines recommend early and consequent treatment of AK^11–14^. At the same time, single lesions may also persist or even regress and clear spontaneously without any active intervention. To circumvent these problems in investigating the progression rates of AK we performed a pooled analysis of efficacy outcomes of the placebo arms of randomized controlled trials (RCT) as a proxy for the regression of AK without any active treatment.

## Methods

### Eligibility criteria

We aimed to investigate adult patients (≥ 18 years of age) with a clinical or histopathological diagnosis of AK. Both immunocompetent, as well as immunocompromised patients such as organ transplant recipients (OTR), were eligible. We only included RCT with an intra- or inter-individual design in which placebo (vehicle cream or gel, hyaluronic acid gel, placebo photodynamic therapy (PDT)) served as a comparison to other active treatments, such as surgical procedures (e.g. excisional biopsies, shave excision), cryosurgery, cryopeeling, ablative laser treatment (e.g. erbium:YAG or carbon dioxide laser), ingenol mebutate 0.015% or 0.05% gel, imiquimod 2.5%, 3.75% or 5% cream, 5-fluorouracil (5-FU) 5% cream, 5-FU 0.5% plus salicylic acid 10% in solution, 3% diclofenac in hyaluronic acid, and PDT with aminolevulinate (ALA) or its ester methyl aminolevulinate (MAL) with illumination from light-emitting diodes or natural daylight. Trials with other designs and trials investigating combination approaches were excluded.

### Outcomes

Outcomes of interest included the participant-specific complete clearance, defined as the number of patients with 100% cleared lesions (dichotomous outcome), and the lesion-specific clearance, defined as the number of cleared lesions compared from baseline to assessment (dichotomous outcome). No restriction regarding the reporting time of the endpoints was set in advance.

### Search strategy and data sources

We searched the electronic databases Medline, Embase (both via Ovid), and the Cochrane library CENTRAL to identify all relevant records until 3rd March 2020 (Supplementary Table 1). Besides, reference lists of included records were screened. No language restrictions were set. The study was not registered a priori. The study protocol can be requested by authors upon reasonable request.

### Study selection

Two authors (TS, AH) independently screened titles and abstracts for eligibility that were identified in the electronic database searches. For records that were considered relevant after the title and abstract screening, full-text articles were obtained and inclusion and exclusion criteria were applied.

### Data collection, synthesis, and management

Information for each included study regarding design, baseline characteristics, type of placebo, as well as outcomes were collected and summarized by two authors independently (TS, AH). Discrepancies were checked by an additional, independent review author (AP, MVH). Data were extracted to an internally piloted data extraction spreadsheet using Microsoft Excel 2016. Wherever possible and suitable, we performed a meta-analysis of quantitative data using R (https://www.r-project.org/) using the packages “meta” and “metafor” to calculate pooled proportions. We used the random-effects model because clinical and methodological heterogeneity between the studies was likely. For all outcomes, the intention-to-treat (ITT) population of the study was used for the analysis. Besides, subgroup analyses were performed regarding (i) the type of placebo (vehicle cream, vehicle gel, placebo PDT or hyaluronic acid), (ii) localization of the lesions (face and scalp versus extremities or trunk), and (iii) immune status (immunocompetent versus immunosuppressed, i.e. OTR). Additionally, subgroup analysis was conducted for (iv) different reported time points after the respective end of treatment.

To address the influence on the pooled effect estimate of single studies, we performed sensitivity analyses by conducting a leave-one-out analysis and identified outliers by creating Baujat plots. The existence of heterogeneity among effect sizes of individual studies was assessed with the I^2^ statistic. Furthermore, we assessed publication bias by creating funnel plot.

## Results

### Study identification

Our initial literature search identified 1625 references. After removing duplicates, 1130 citations remained. Following title and abstract screening, 1066 references were not considered relevant because they did not meet the inclusion criteria and were consequently excluded accordingly. Hence, 64 records underwent full-text review (Supplementary Fig. 1). Of these 64 records, 26 were dismissed since they reported inappropriate outcomes (n = 5), did not provide any results (n = 4), did not match the eligibility criteria regarding the study design (n = 6), or since they were duplicates (n = 11). Finally, 38 records met the eligibility criteria and were included in the pooled analysis (Supplementary Table 2). Three of the studies reported data on OTR while the remaining studies investigated immunocompetent patients.

### Participant-specific regression

**Figure 1:**
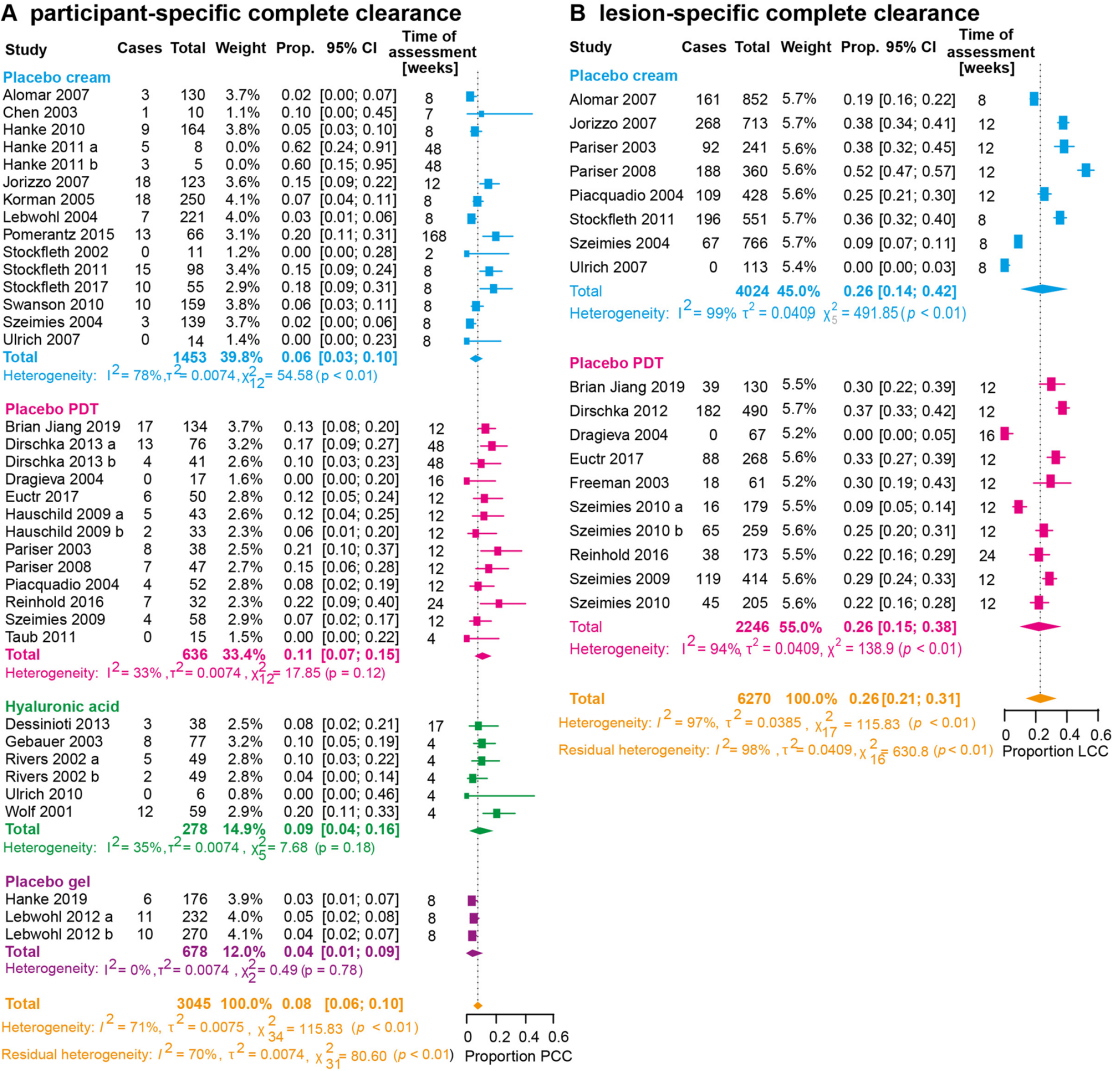
(**A**) Forest plot examining the pooled proportion of the adjusted overall participant-specific complete clearance (PCC) rate for the different types of placebos. Outliers have been excluded. (**B**) Forest plot examining the pooled proportion of the adjusted overall lesion-specific complete clearance rate and the lesion-specific clearance rate for the different types of placebos. In all cases, forest plots examining single-armed trials are shown. Random-effects analysis was used. The diamond represents the estimate from the study. The width of the line extending from each diamond represents the 95% confidence interval (CI). Prop.: proportion.

Data on participant-specific complete clearance rates was available from 37 placebo arms originating from 34 RCT including an overall sample size of n = 3045 patients. The Baujat plot revealed 2 outliers (2 placebo arms from 1 study) in the sensitivity analysis^15^. After removal, the pooled participant-specific clearance rate was calculated to be 8% (95% CI 6–10%, I^2^ = 71%). When stratified according to the type of placebo, placebo PDT yielded the highest pooled estimate with 11% (95% CI 7–15%, I^2^ = 33%), followed by hyaluronic acid (9%, 95% CI 4–16%, I^2^ = 35%) and vehicle cream (6%, 95% CI 3–10%, I^2^ = 78%). Vehicle gel used as a placebo intervention revealed a pooled clearance rate of 4% (95% CI 1–9%, I^2^ = 0%) (Fig. 1A).

The evidence identified enabled a subgroup analysis regarding the localization of AK. Patients with AK on the face and/or scalp showed a higher pooled clearance rate with 9% (95% CI 6–12%, I^2^ = 79%) than patients with AK on the trunk or extremities (6%, 95% CI 1–14%, I^2^ = 76%) (Supplementary Fig. 2a).

Data was available for the subgroup OTR in 3 placebo arms from 3 studies^16–18^. Overall, the participant-specific clearance rate was estimated to be 0% (95% CI 0–7%, I^2^ = 0%) in immunosuppressed patients in comparison to 8% (95% CI 6–11%, I^2^ = 72%) in immunocompetent patients.

**Figure 2:**
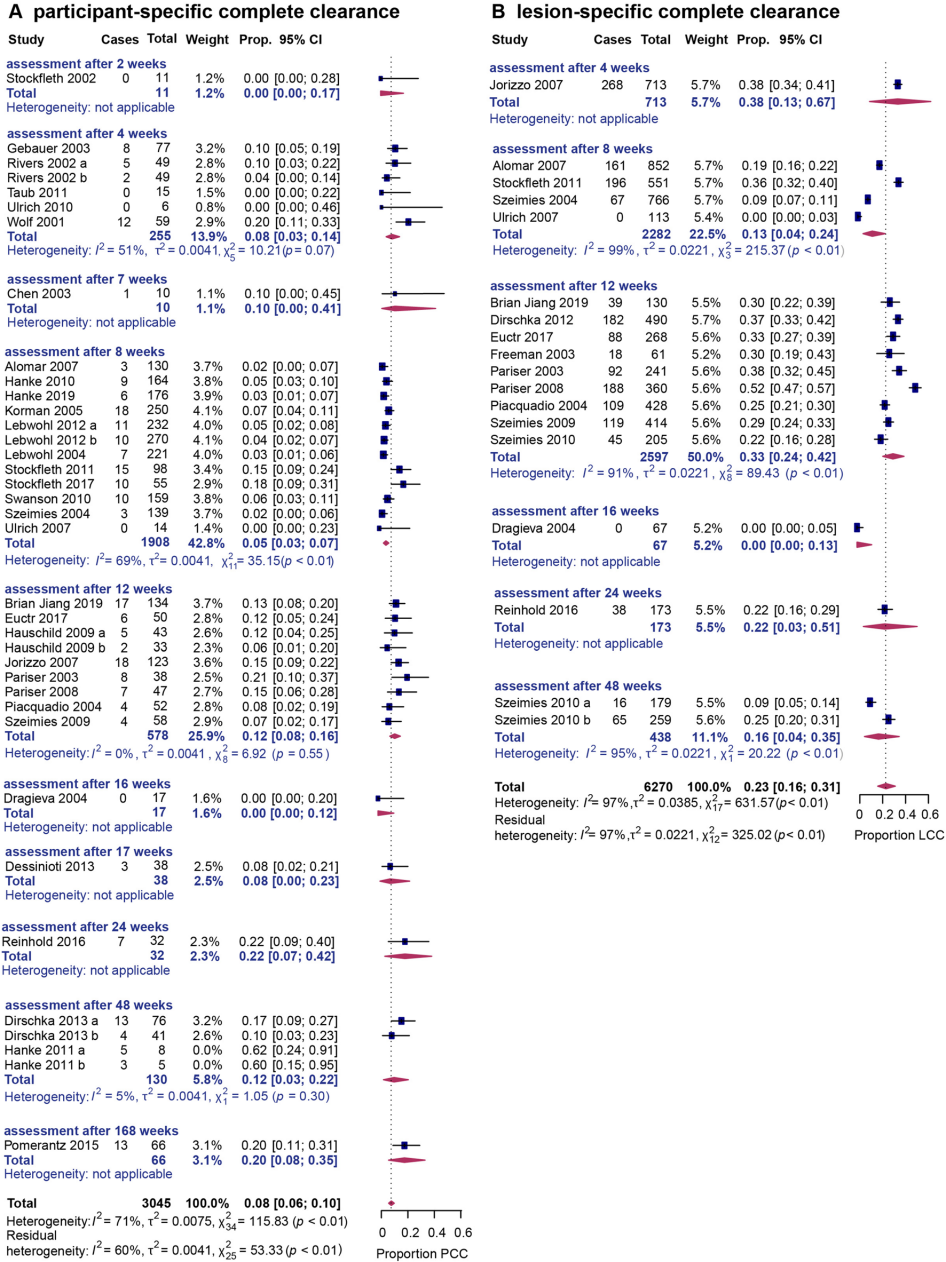
(**A**) Forest plot examining the pooled proportion of the outcome participant-specific complete clearance (PCC) rate for the different time points. Outliers have been excluded. (**B**) Forest plot examining the pooled proportion of the outcome lesion-specific complete clearance (LCC) rate for the different time points. In all cases, forest plots examining single-armed trials are shown. Random-effects analysis was used. The diamond represents the estimate from the studies. The width of the line extending from each diamond represents the 95% confidence interval (CI). Prop.: proportion.

Additionally, subgroup analysis was performed for distinct time points of assessment for this outcome. Four weeks after the end of treatment, the participant-specific clearance rate was 8% (95% CI 3–14%, I^2^ = 51%), while after 8 weeks it was estimated to be 5% (95% CI 3–7%, I^2^ = 69%) (Fig. 2A). Interestingly, this rate increased to 12% after 12 weeks (95% CI 8–16%, I^2^ = 0%) and 48 weeks (95% CI 3–22%, I^2^ = 5%), respectively. No clear trend was observed in studies that reported this outcome at several distinct time points (Supplementary Fig. 5a)^19–21^. The funnel plot showed a slight asymmetry with some risk for publication bias (p = 0.043) for this outcome (Supplementary Fig. 3a).

### Lesion-specific regression

Lesion-specific clearance rates were reported from 18 placebo arms of 18 RCT including n = 5730 lesions. No outliers were identified when examining the Baujat plot in the sensitivity analysis. The pooled lesion-specific clearance rate was 23% (95% CI 16–31%, I^2^ = 97%) (Fig. 1B). Data regarding the type of placebo could only be retrieved for vehicle cream and placebo PDT. The lesion-specific clearance rates were similar with 24% (95% CI 13–37%, I^2^ = 99%) after vehicle cream and 22% (95% CI 12–33%, I^2^ = 94%) after placebo PDT. According to their localization, AK lesions on the trunk or extremities showed a pooled lesion-specific clearance rate of 30% (95% CI 3–69%). However, data were only available from one placebo arm^22^. AK located on the face and/or scalp had a pooled clearance rate of 25% (95% CI 17–36%, I^2^ = 98%) (Supplementary Fig. 4a).

Additionally, data for the subgroup OTR was available from 2 studies revealing lesion-specific clearance rates of 0% (95% CI 0–5%, I^2^ = 0%) in comparison to 28% (95% CI 22–33%, I^2^ = 97%) in immunocompetent patients (Supplementary Fig. 4b)^16,17^.

When investigating the lesion-specific clearance rates at different time points, 13% of lesions were cleared 8 weeks after the end of treatment (95% CI 4–24%, I^2^ = 99%), while 33% were cleared after 12 weeks (95% CI 24–42%, I^2^ = 91%) (Fig. 2B).

In studies assessing the lesion-specific clearance at different time points, a slightly decreasing trend with time was observed in 4 trials^21,23,24^. In contrast, the lesion-specific clearance rate increased during follow-up in one trial (Supplementary Fig. 5b)^22^. There was no evidence for publication bias for this outcome (Supplementary Fig. 3b).

## Discussion

There has been an intensive, long-standing, and still ongoing debate on whether each AK warrants active treatment or not. To accurately investigate the natural course and history of AK poses a major challenge owing to the scarcity of high-quality studies assessing a longer follow-up of at least 6–12 months and the high heterogeneity of the populations studied. Besides, AK and adjacent field cancerization is a chronic skin disease in which multiple intervening therapies over long follow-up periods may substantially influence the disease course and progression rates of AK. International treatment guidelines for AK recommend early and consequent treatment, as it is currently not reliably possible to predict whether and when a lesion will progress into invasive SCC^13,14^. Real-world data investigating the underlying motivation of patients undergoing AK treatment also confirms that most patients decide for treatment to avoid the transition to invasive SCC and since AK are widely considered precancerous lesions^25^. To this end, it is surprising how little information on patient-reported outcmes and patient perspectives in AK treatment is yet available^26^. Despite this, the transformation risk is presumably low for single lesions as progression rates have been estimated to range between 0 and 0.075% per lesion-year^8^. However, the risk of malignant conversion increases rapidly when multiple AK or field cancerization are present^9,10^. On the other hand, spontaneous remission rates of 15% to 63% after one year have been reported previously so the question inevitably arises whether each AK should be treated or whether watchful waiting is also justified in a low-risk situation^9,11^. Until now, published data on spontaneous remission rates of AK are heterogeneous. Regression of AK has been reported in studies investigating the application of daily sunscreen in both immunocompetent and immunosuppressed patients in comparison to patients who do not use sunscreen^27–30^. Overall, previous research suggests that the probability for the regression of solitary lesions ranges between 15 and 63% after 1 year^9^.

Here, we performed a pooled analysis of the participant- and lesion-specific clearance rates of the placebo arms of RCT as a proxy for the spontaneous regression of AK. Overall, the pooled participant-specific clearance rate was calculated to be 8% and the lesion-specific clearance rate to be 23% in the random-effects model. This highlights that only 8% of patients investigated in this collective will achieve complete healing of all AK without any active treatment. However, on a lesion-specific level, every single lesion investigated in the analysis has only a chance of less than 25% regressing without any active treatment.

Nevertheless, the findings underline that disease control—especially when the ultimate target is to clear all AK and not just single lesions—may not be achieved by pursuing the strategy of watchful waiting and implies that additional treatment is advised. Nowadays, a wide range of lesion- as well as field-directed approaches is available for the effective and safe treatment of AK^14^. Approaches may even be combined to synergize the strengths of the individual treatment regimens and to overcome their barriers^31–35^.

Notably, OTR did not show any regression of their AK neither for the outcome participant-specific clearance rate nor for the outcome lesion-specific clearance rate. This is in line with previous research suggesting that the natural course of AK is less favorable in immunocompromised individuals with a higher likelihood of conversion to cSCC and lower rates of spontaneous regression^36^. Thus, our findings emphasize that watchful waiting is contraindicated for this vulnerable subgroup and that OTR with AK should always be advised to undergo consequent treatment. Interestingly, a recent systematic review found that there is surprisingly little evidence on how to best treat AK in OTR but concluded that PDT is among the most favorable interventions for OTR^37^. Moreover, regression rates are even less favorable in OTR who spent > 50% of a typical weekday in the sun compared to those who spent minimal time in the sun during a typical weekday^38^. Furthermore, age, parents’ country of origin, hair color, skin cancer history, and recent AK treatment were significantly associated with progression^38^. Hence, this patient collective requires increased attention and should be educated about appropriate sun protection and preventive measures such as skin cancer screening programs or chemoprevention like the intake of nicotinamide or acitretin^39–43^.

Interestingly, placebo PDT showed the highest participant-specific complete clearance rates with 11% in comparison to the other vehicle types. This might be explained by the fact that PDT is usually performed in an office-based setting involving pretreatment of the skin with curettage to remove scaling, whereas a placebo cream or gel is self-applied by the patient without physical pretreatment. Thus, this effect of curettage might bias the results. Certainly, the concept of the placebo effect plays an important role which cannot be neglected^44,45^. Additionally, vehicle gel of diclofenac revealed participant-specific complete clearance rates of 9%. However, this type of vehicle still contains an active ingredient, i.e. hyaluronic acid, which has been shown to improve wound healing^46–48^. Thus the regression rates remain questionable whether they might have been induced by the active substance or whether a spontaneous regression of all lesions occurred. Besides this, as AK are mostly diagnosed clinically and biopsies are rarely taken, the possibility of a misdiagnosis of a common inflammation, that heals without any therapy, should also be kept in mind when interpreting the results.

Importantly, the results from this analysis do not allow any conclusions on whether the AK will remain to be cleared or whether the AK will recur or eventually progress to invasive cSCC. A recent pooled analysis found the long-term participant-specific recurrence rates for the placebo arms of RCT after at least one year of follow-up to be 44%, and, thus lower than the observed participant-specific clearance rates for diclofenac (85%), ablative laser treatments (64%) and 5-FU (52%)^49^. Notably, lesion-specific recurrence rates were estimated to be lowest for placebo with 15%, followed by PDT^49^. Despite this, we assume that the risk of progression outweighs the chance for spontaneous regression, especially in high-risk subgroups such as OTR. Besides this, our results might be affected by bias arising in the RCTs assessing the placebo effect on AK. Histopathological outcomes are rarely reported in RCTs and especially after therapy, a biopsy is performed rarely when a lesion has cleared clinically. Hence, in absence of histological analysis, it is difficult to confirm the spontaneous regression of AK. Clinical assessment is observer-dependent and may be difficult for physicians who can misdiagnose a complete response in those AK in which scaling and redness have improved with placebo. Furthermore, the sole application of vehicle creams and moisturizers can result in a better clinical appearance of AK without real, i.e. histologically assessed improvement. Thus, our results have to be interpreted with caution. Certainly, not assessing the histopathological complete and lesion-specific clearance is an important limitation of this study.

In general, the approach employed here enables the incorporation of the participant- and lesion-specific clearances rates from the highest possible evidence, i.e. RCT. Besides, using the results from such trials guarantees that no intercurrent treatments skew the results as pivotal trials and RCT usually require a washout period and do not allow any intercurrent treatments. However, limitations of this pooled analysis include the clinical as well as statistical heterogeneity of the included placebo arms from the respective RCT for nearly all outcomes and subgroup analyses. However, by creating Baujat plots and excluding outliers we decreased statistical heterogeneity, whenever feasible. Besides, the funnel plot for the outcome participant-specific clearance showed that publication bias is likely. Indeed, we did not perform a systematic review and did not search for grey literature such as trial registers or conference abstracts and instead used medical databases. Thus, these important methodological deficits limit the generalizability of our findings. Nevertheless, more high-quality trials should be performed in the future to investigate both the potential of regression as well as the potential of progression of AK to cSCC. Besides, we conclude that there is an urgent need for better classification systems to predict the negative outcome of transformation to aggressive cSCC.

## Supplementary Information

Supplementary Figure 1.

Supplementary Figure 2.

Supplementary Figure 3.

Supplementary Figure 4.

Supplementary Figure 5.

Supplementary Figure Legends.

Supplementary Table 1.

Supplementary Table 2.

## Data Availability

The data that support the findings of this study are available from the corresponding author upon reasonable request.
