# Supplementary figures and images for: Spontaneous regression rates of actinic keratosis: a systematic review and pooled analysis of randomized controlled trials
Source: Sci Rep. 2022 Apr 7;12:5884. doi: 10.1038/s41598-022-09722-8 (PMC8990007; doi:10.1038/s41598-022-09722-8)

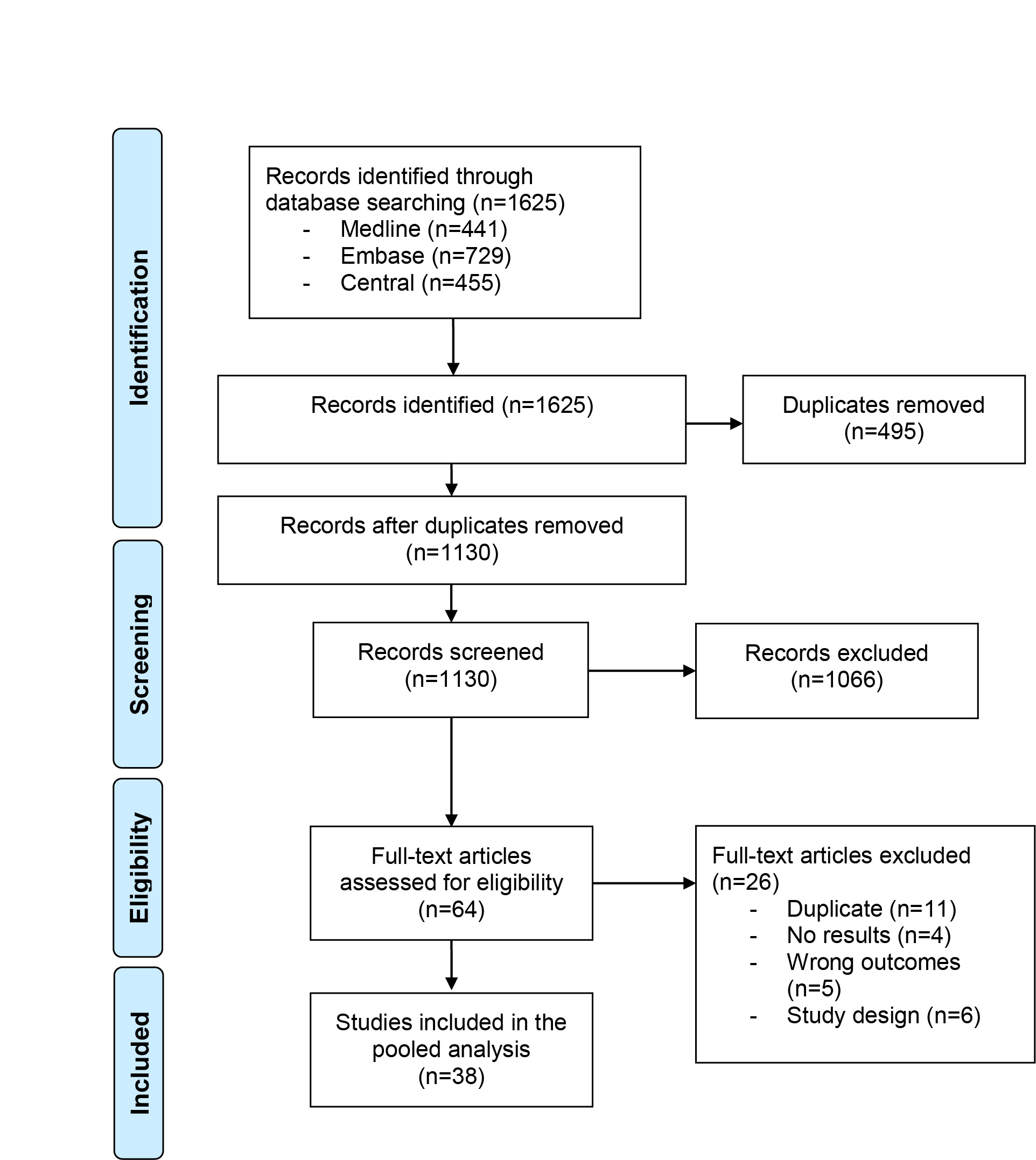

Supplement: Supplementary file 1 — Supplementary Figure 1. [file 41598_2022_9722_MOESM1_ESM.jpg]

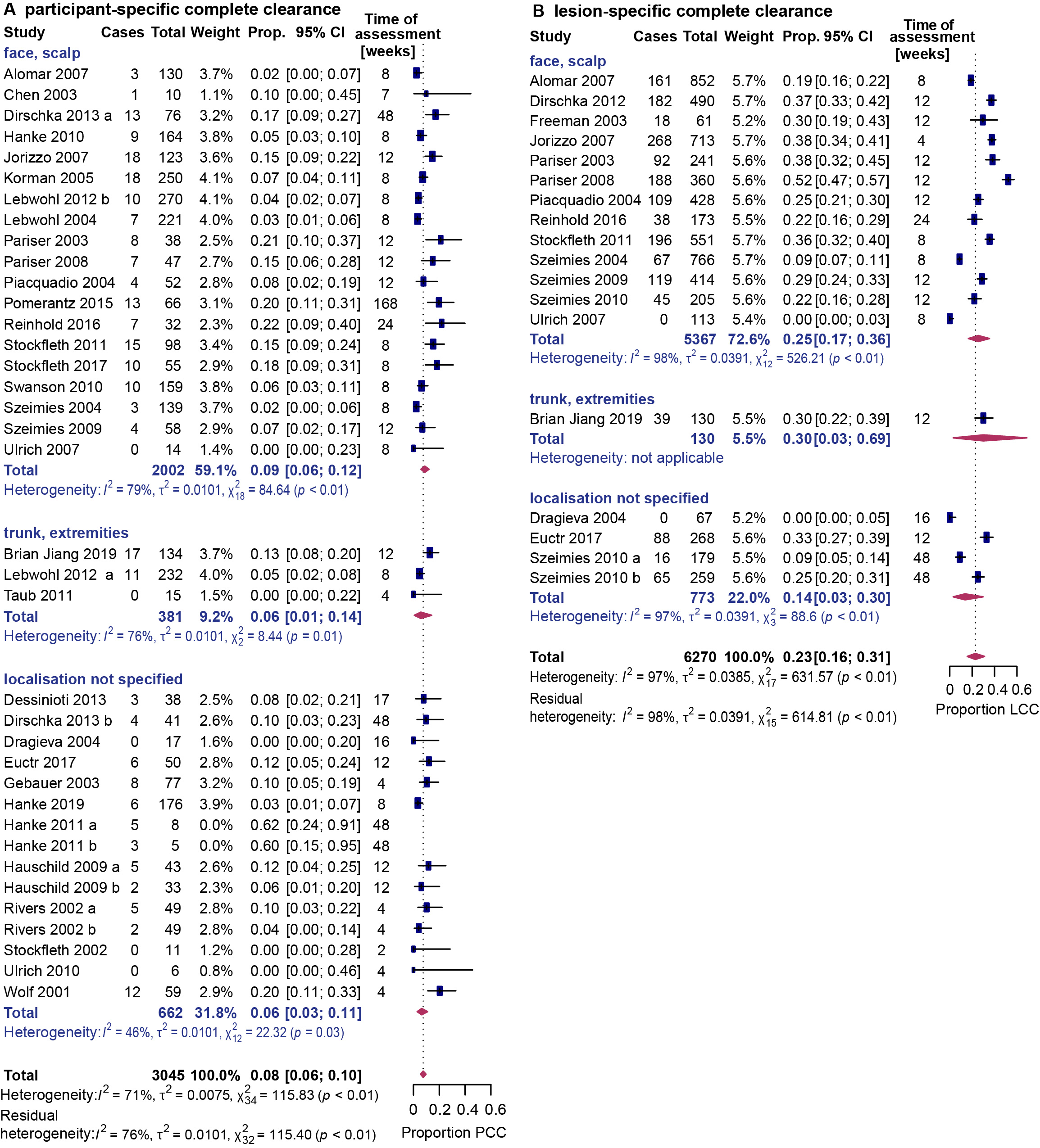

Supplement: Supplementary file 2 — Supplementary Figure 2. [file 41598_2022_9722_MOESM2_ESM.jpg]

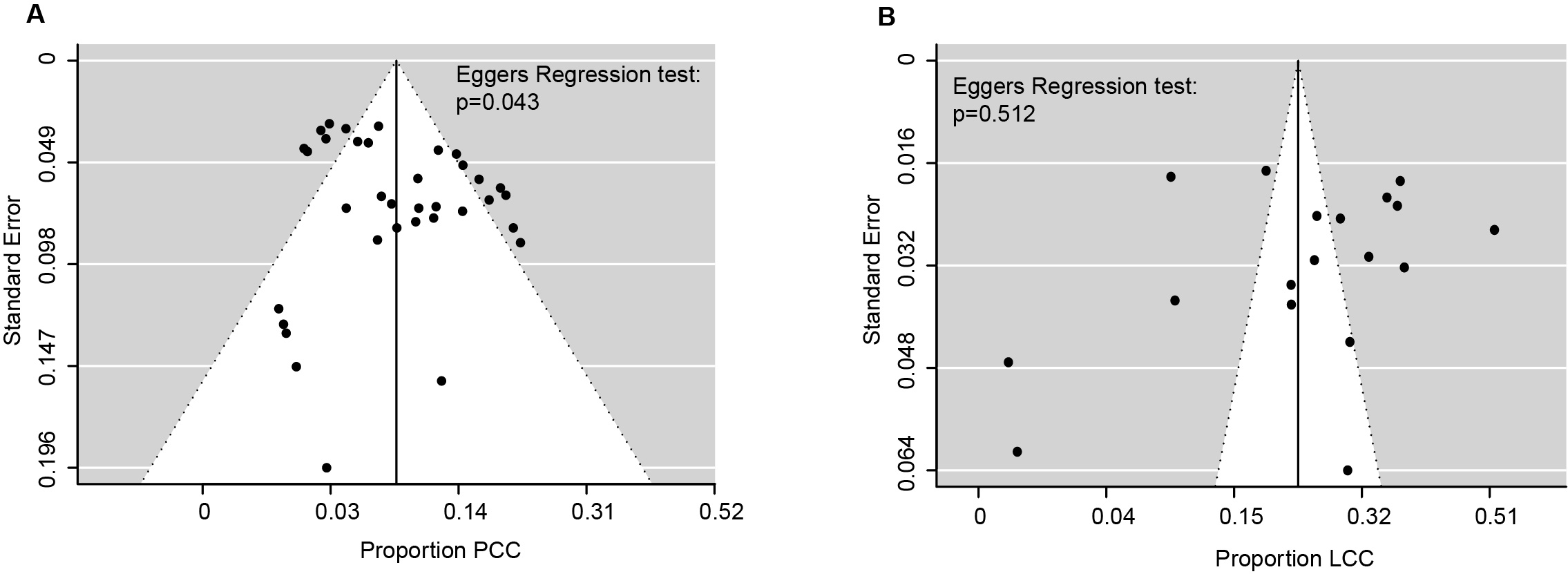

Supplement: Supplementary file 3 — Supplementary Figure 3. [file 41598_2022_9722_MOESM3_ESM.jpg]

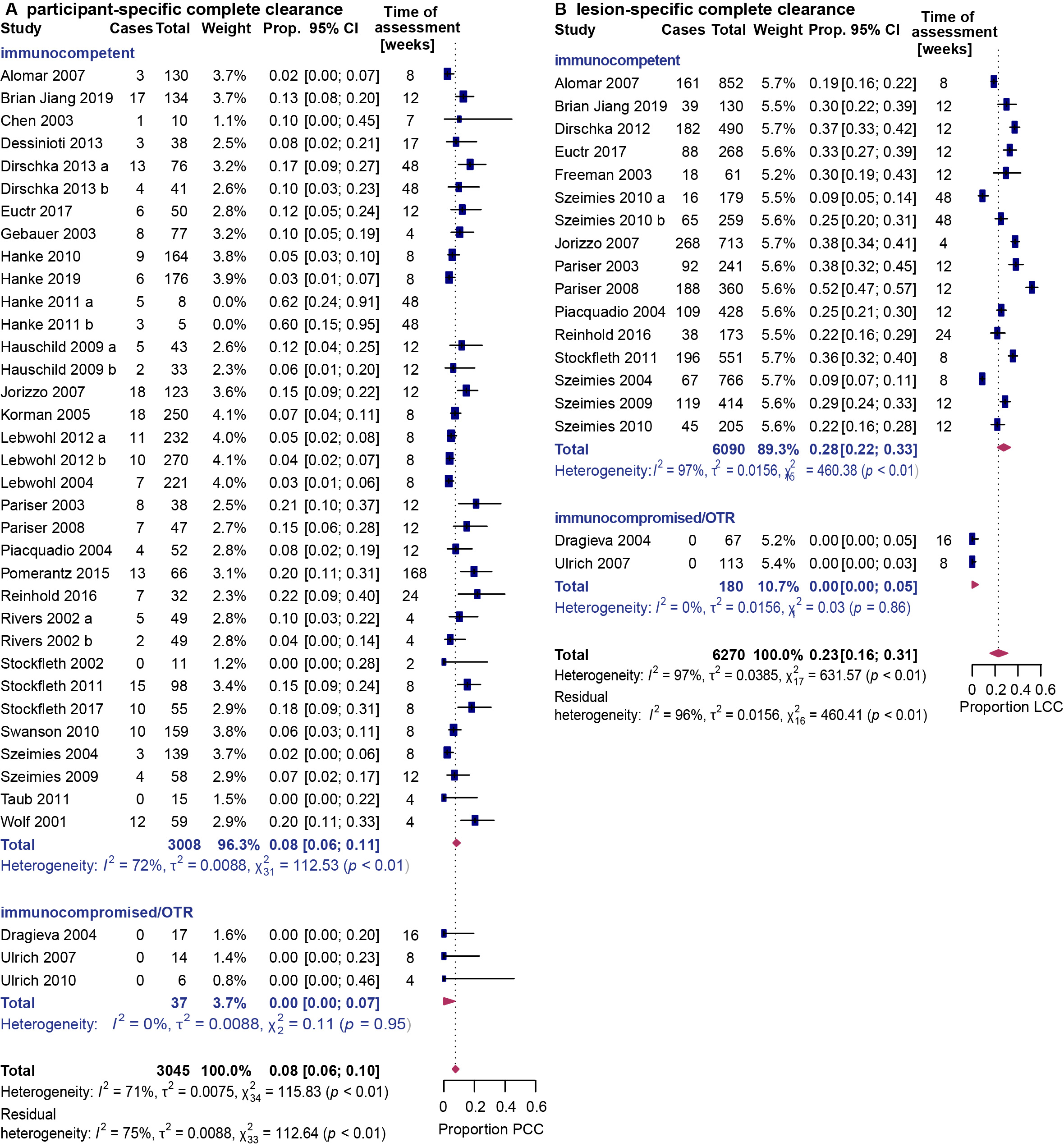

Supplement: Supplementary file 4 — Supplementary Figure 4. [file 41598_2022_9722_MOESM4_ESM.jpg]

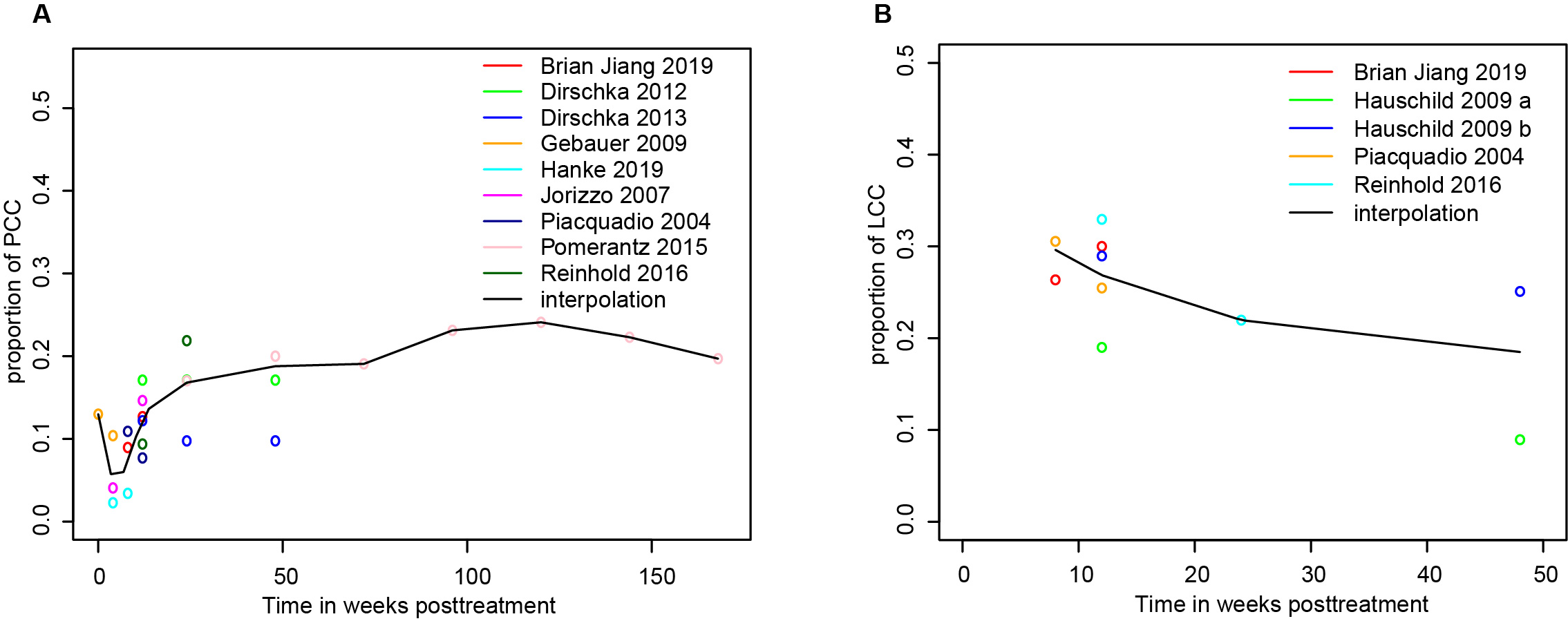

Supplement: Supplementary file 5 — Supplementary Figure 5. [file 41598_2022_9722_MOESM5_ESM.jpg]
