# Supplementary material for: Spontaneous regression rates of actinic keratosis: a systematic review and pooled analysis of randomized controlled trials
Source: Sci Rep. 2022 Apr 7;12:5884. doi: 10.1038/s41598-022-09722-8 (PMC8990007; doi:10.1038/s41598-022-09722-8)
Supplement: Supplementary file 6 — Supplementary Figure Legends. [file 41598_2022_9722_MOESM6_ESM.docx]

**Supplementary Figure 1:** Flow chart of the literature identification process according to the PRISMA guidelines.

**Supplementary Figure 2:** a) Forest plot examining the pooled proportion of the outcome participant-specific clearance (PCC) rate for the different localizations of the AK. Outliers have been excluded. b) Forest plot examining the pooled proportion of the outcome lesion-specific clearance (LCC) rate stratified by immunocompromised (organ transplant recipients, OTR) and immunocompetent patients. Outliers have been excluded.

In all cases, forest plots examining single-armed trials are shown. Random-effects analysis was used. The diamond represents the estimate from the study. The width of the line extending from each diamond represents the 95 % confidence interval (CI). Prop.: proportion.

**Supplementary Figure 3:** Funnel plot for the assessment of publication bias for the outcome a) participant-specific clearance (PCC) rate and b) lesion-specific clearance (LCC) rate.

**Supplementary Figure 4:** a) Forest plot examining the pooled proportion of the outcome participant-specific clearance (PCC) rate for the different localizations of AK. b) Forest plot examining the pooled proportion of the outcome lesion-specific clearance (LCC) rate stratified by immunocompromised (organ transplant recipients, OTR) and immunocompetent patients. Prop.: proportion.

**Supplementary Figure 5:** a) Participant-specific clearance (PCC) rates in time. The circles represent one clearance rate at a certain time point of one study (studies are color-coded). The black line shows the interpolation line between the mean of clearance rates at each time point which depicts the overall trend in time. b) Lesion-specific clearance (LCC) rates in time. The circles represent one clearance rate at a certain time point of one study (studies are color-coded). The black line shows the interpolation line between the mean of clearance rates at each time point which depicts the overall trend in time.
