# Supplementary material for: Spontaneous regression rates of actinic keratosis: a systematic review and pooled analysis of randomized controlled trials
Source: Sci Rep. 2022 Apr 7;12:5884. doi: 10.1038/s41598-022-09722-8 (PMC8990007; doi:10.1038/s41598-022-09722-8)
Supplement: Supplementary file 7 — Supplementary Table 1. [file 41598_2022_9722_MOESM7_ESM.docx]

**Supplementary Table 1: Search string for the literature identification in the medical databases A) Medline, B) Embase, and C) the Cochrane Library.**

| **Ovid MEDLINE(R) and Epub Ahead of Print, In-Process & Other Non-Indexed Citations, Daily and Versions(R) 1946 to March 02, 2020**  N=441 (03.03.2020) |
| --- |
| 1. actinic keratosis.mp. or exp Keratosis, Actinic/  2. exp Skin Neoplasms/ or solar keratosis.mp.  3. senile keratosis.mp.  4. field change.mp.  5. actinically damaged field.mp.  6. exp Precancerous Conditions/ or field-cancerized.mp.  7. 1 or 2 or 3 or 4 or 5 or 6  8. clinical trial.mp. or exp Clinical Trial/  9. randomized controlled trial.mp. or exp Randomized Controlled Trial/  10. 8 or 9  11. placebo.mp. or exp Placebo Effect/  12. vehicle.mp.  13. 11 or 12  14. 7 and 10 and 13 |
| **Embase** 1974 to 2020 March 02  N=729 (03.03.2020) |
| 1. actinic keratosis.mp. or actinic keratosis/  2. solar keratosis.mp.  3. senile keratosis.mp.  4. field change.mp.  5. actinically damaged field.mp.  6. field-cancerized.mp. or exp precancer/  7. actinic keratoses.mp.  8. 1 or 2 or 3 or 4 or 5 or 6 or 7  9. randomized controlled trial.mp. or exp randomized controlled trial/  10. clinical trial.mp. or exp clinical trial/  11. exp placebo/ or placebo.mp.  12. vehicle.mp.  13. controlled study.mp. or exp controlled study/  14. 9 or 10 or 13  15. 11 or 12  16. 8 and 14 and 15 |
| **Cochrane Library CENTRAL**  **N=455 trials (03.03.2020)** |
| ID Search Hits  #1 actinic keratos* 996  #2 solar keratos* 68  #3 senile keratos* 5  #4 MeSH descriptor: [Keratosis, Actinic] explode all trees 382  #5 MeSH descriptor: [Precancerous Conditions] explode all trees 1592  #6 field cancerization 78  #7 #1 or #2 or #3 or #4 or #5 or #6 2217  #8 vehicle 7329  #9 placebo 308412  #10 dummy 6768  #11 #8 or #9 or #10 316354  #12 randomized controlled trial 965181  #13 trial 1196504  #14 #12 and #13 965181  #15 #7 and #11 and #14 483 |
