# Supplementary material for: Spontaneous regression rates of actinic keratosis: a systematic review and pooled analysis of randomized controlled trials
Source: Sci Rep. 2022 Apr 7;12:5884. doi: 10.1038/s41598-022-09722-8 (PMC8990007; doi:10.1038/s41598-022-09722-8)
Supplement: Supplementary file 8 — Supplementary Table 2. [file 41598_2022_9722_MOESM8_ESM.docx]

**Supplementary Table 2: Baseline characteristics of the included randomized controlled trials.**

| **Study** | **Sample size of the placebo arm** | **Type of placebo** | **Active intervention** | **Localization of AK** | **OTR** |
| --- | --- | --- | --- | --- | --- |
| Alomar 2007 | 130 | vehicle cream | Imiquimod cream 5% | balding scalp,face | no |
| Brian Jiang 2019 | 134 | vehicle PDT | ALA-PDT | one upper extremity (dorsal hand or forearm) | no |
| Chen 2003 | 11 | vehicle cream | Imiquimod cream 5% | scalp, forehead, temples, or both cheeks | no |
| Dessinioti 2013 | 38 | vehicle cream | Diclofenac natrium 3% in hyaluronic acid 2.5% gel | not reported | no |
| Dirschka 2012 (ALA AK CT002) | 76 | vehicle PDT | ALA-PDT | face/forehead, scalp | no |
| Dragieva 2004 | 17 | vehicle PDT | MAL-PDT | face/scalp, extremities | kidney: 13, heart: 4 |
| EudraCT Number: 2017-000486-72 (ALA-AK-CT010) | 50 | vehicle PDT | ALA-PDT | trunk/neck, extremities | no |
| Freeman 2003 | 23 | vehicle PDT | MAL-PDT | face,scalp | no |
| Gebauer 2003 | 77 | hyaluronic acid | Diclofenac natrium 3% in hyaluronic acid 2.5% gel | head/neck, hands, arms | no |
| Hanke 2010 | 164 | vehicle cream | Imiquimod cream 2.5% | face,scalp | no |
|  |  |  | Imiquimod cream 3.75% |  |  |
| Hanke 2019 | 177 | vehicle gel | Ingenol mebutate gel 0.027% | face, balding scalp, chest | no |
| Hanke 2011 (2 week cycle) | 8 | vehicle cream | Imiquimod cream 2.5% | Face and other | no |
|  |  |  | Imiquimod cream 3.75% |  |  |
| Hanke 2011 (3 week cycle) | 6 | vehicle cream | Imiquimod cream 2.5% | face | no |
|  |  |  | Imiquimod cream 3.75% |  |  |
| Hauschild 2009 AK 03 | 34 | vehicle PDT | ALA-PDT | forehead, scalp, cheek, nose, ear, other | no |
| Hauschild 2009 AK 04 | 49 | vehicle PDT | ALA-PDT | forehead, scalp, cheek, nose, ear, other | no |
| Jorizzo 2007 | 123 | vehicle cream | Imiquimod cream 5% | balding scalp,face | no |
| Korman 2005 | 250 | vehicle cream | Imiquimod cream 5% | balding scalp,face | no |
| Lebwohl 2012 | 270 | vehicle gel | Ingenol mebutate gel 0.015% | face,scalp | no |
| Lebwohl 2012 | 232 | vehicle gel | Ingenol mebutate gel 0.05% | trunk, extremities | no |
| Lebwohl 2004 | 221 | vehicle cream | Imiquimod cream 5% | face,balding scalp | no |
| Pariser 2003 | 38 | vehicle PDT | MAL-PDT | face, scalp | no |
| Pariser 2008 | 47 | vehicle PDT | MAL-PDT | face, scalp | no |
| Piacquadio 2004 | 62 | vehicle PDT | ALA-PDT | face,scalp | no |
| Pomerantz 2015 | 464 | vehicle cream | 5-Fluorouracil cream 5% | face and ear | no |
| Reinhold 2016 | 32 | vehicle PDT | ALA-PDT | face and forehead, bald scalp | no |
| Rivers 2002 (30 day cycle) | 49 | hyaluronic acid | Diclofenac natrium 3% in hyaluronic acid 2.5% gel | forehead, central face, scalp, dorsal hands | no |
| Rivers 2002 (60 day cycle) | 49 | hyaluronic acid | Diclofenac natrium 3% in hyaluronic acid 2.5% gel | forehead, central face, scalp, dorsal hands | no |
| Stockfleth 2002 | 11 | vehicle cream | Imiquimod cream 5% | scalp, forehaead, dorsal forearm, neck, dorsal hands | no |
| Stockfleth 2011 | 98 | vehicle cream | 5-Fluorouracil 0.5% and salicylic acid 10.0% | scalp, face | no |
|  |  |  | Diclofenac natrium 3% in hyaluronic acid 2.5% gel |  |  |
| Stockfleth 2017 | 55 | vehicle cream | 5-Fluorouracil 0.5% and salicylic acid 10.0% | scalp, face/forehead | no |
| Swanson 2010 | 159 | vehicle cream | Imiquimod cream 2.5% | face,scalp | no |
|  |  |  | Imiquimod cream 3.75% |  |  |
| Szeimies 2004 | 139 | vehicle cream | Imiquimod cream 5% | face,scalp | no |
| Szeimies 2009 | 58 | vehicle PDT | MAL-PDT | face, scalp | no |
| Szeimies 2010 | 41 | vehicle PDT | ALA-PDT | face and/or scalp | no |
| Taub 2011 | 15 | vehicle PDT | ALA-PDT | hands, arms | no |
| Ulrich 2007 | 14 | vehicle cream | Imiquimod cream 5% | face,scalp | liver: 2, kidney: 10, heart: 2 |
| Ulrich 2010 | 8 | hyaluronic acid | Diclofenac natrium 3% in hyaluronic acid 2.5% gel | face, scalp, hands | liver: 1, kidney: 5, heart: 2 |
| Wolf 2001 | 59 | hyaluronic acid | Diclofenac natrium 3% in hyaluronic acid 2.5% gel | forehead, central face, scalp, arms, hands | no |

**Abbreviations**: ALA-PDT = photodynamic therapy with aminolevulinate; MAL-PDT = photodynamic therapy with methyl aminolevulinate; OTR = organ transplant recipient; PDT = photodynamic therapy.
